# Molecular epidemiology of chicken anaemia virus in sick chickens in China from 2014 to 2015

**DOI:** 10.1371/journal.pone.0210696

**Published:** 2019-01-18

**Authors:** Shuai Yao, Tianbei Tuo, Xiang Gao, Chunyan Han, Nana Yan, Aijing Liu, Honglei Gao, Yulong Gao, Hongyu Cui, Changjun Liu, Yanping Zhang, Xiaole Qi, Altaf Hussain, Yongqiang Wang, Xiaomei Wang

**Affiliations:** 1 College of Veterinary Medicine, Northeast Agricultural University, Harbin, China; 2 Division of Avian Infectious Diseases, State Key Laboratory of Veterinary Biotechnology, Harbin Veterinary Research Institute, Chinese Academy of Agricultural Sciences, Harbin, China; 3 College of Wildlife Resource, Northeast Forestry University, Harbin, China; Sun Yat-Sen University, CHINA

## Abstract

Chicken anaemia virus (CAV), a member of the genus *Gyrovirus*, is the etiological agent of chicken infectious anaemia. CAV infects bone marrow-derived cells, resulting in severe anaemia and immunosuppression in young chickens and a compromised immune response in older birds. We investigated the molecular epidemiology of CAV in sick chickens in China from 2014 to 2015 and showed that the CAV-positive rate was 13.30%, in which mixed infection (55.56%) was the main type of infection. We isolated and identified 15 new CAV strains using different methods including indirect immunofluorescence assay and Western Blotting. We used overlapping polymerase chain reaction to map the whole genome of the strains. Phylogenetic analyses of the obtained sequences and related sequences available in GenBank generated four distinct groups (A–D). We built phylogenetic trees using predicted viral protein (VP) sequences. Unlike CAV VP2s and VP3s that were well conserved, the diversity of VP1s indicated that the new strains were virulent. Our epidemiological study provided new insights into the prevalence of CAV in clinical settings in recent years in China.

## Introduction

The genus Gyrovirus, a diverse group of non-enveloped icosahedral viruses containing circular single-stranded DNA [1], infects a wide range of hosts. They also trigger several serious diseases in animals as causative agents. In particular, chicken anaemia virus (CAV), a member of family *Anelloviridae* genus *Gyrovirus*, is the etiological agent of chicken infectious anaemia [2]. CAV infects several bone marrow-derived cells which results in severe anaemia and immunosuppression in young chickens. In terms of older birds, CAV can jeopardize the immune responses [3, 4]. Since its first reported in 1979 [2], CAV infection has become epidemic among chicken populations on a global scale [5–7]. CAV also has dramatic financial impact in areas of intensive chicken farming. Vaccination is generally used to contain the spread of the virus [8]. In a recent study, a novel human gyrovirus was isolated from a skin swab and designated as human *Gyrovirus* (HGyV) [9]. Since Circovirus shares partial homology to CAV, the identification of HGyV signals possible threats for human pathogenesis, further investigation is yet required.

The negative-sense CAV genome consists of 2,319 nucleotides and is replicated by a rolling-circle mechanism; but the packaging and egress of viral particles are poorly characterised [1, 10]. The CAV genome encodes multiple overlapping open reading frames (ORFs) [11] that are translated into three main distinct polypeptides: CAV viral protein 1 (VP1, 52 kDa), viral protein 2 (VP2, 24 kDa) and viral protein 3 (VP3, 16 kDa). VP1 is the major structural protein while the VP2 is a replicase with dual-specificity phosphatase activity [12]. VP3, also named apoptin, is also a non-structural protein that mainly implicats in the induction of apoptosis and viral cytotoxicity in host cells.

In 1996, CAV was first reported from young broilers in China [13]. 42% of overall seroprevalence was shown in farms of five Chinese provinces in a domestic poultry survey [14]. In addition, a high prevalence of 87% resulted in studies of the virus on live bird markets in Southeast China [15].

In the present study, our group investigated the epidemiology of CAV in sick or dead chickens in 12 provinces throughout China from 2014 to 2015. Totally, we obtained 96 positive results for CAV infection in 722 clinical samples, 24 out of 149 in 2014, and 72 out of 573 in 2015. We analysed the infection type of CAV in association with other pathogens including Marek’s disease virus (MDV), reticuloendotheliosis virus (REV), avian leukosis virus (ALV), avian gyrovirus 2 (AGV2), and avian reovirus (ARV). We found that coinfection was the main infection type of CAV. In addition, we analysed the characteristics of the new CAV sequenced strains together with those available in GenBank. The analysis revealed that all the sequences could be clustered into four major groups. Furthermore, we compared the key amino acids in VP1 that determined the virulence of CAV, providing new insights into the epidemiology of CAV.

## Materials and methods

### Ethics statement

All applicable international, national, and/or institutional guidelines for the care and use of animals were followed. The animal experiments were performed in strict compliance with the Guideline for the Care and Use of Laboratory Animals of the Ministry of Science and Technology of the People’s Republic of China. The Committee of the Ethics of Animal Experiments at the Harbin Veterinary Research Institute (HVRI) of the Chinese Academy of Agricultural Sciences (CAAS) approved the animal experiment protocols. A permission from China Agriculture Research System was issued for the field studies, and all the owners of the land or farms were informed consent to conduct the study on this site.

### Sample information

In total, 722 clinical specimens from suspected sick fowl or fowl embryos (mostly comprising livers, spleens, and thymuses) were collected from 2014 to 2015, covering many provinces of China, including Heilongjiang (235 samples), Jilin (122 samples), Liaoning (109 samples), Shanghai (23 samples), Shanxi (11 samples), Hebei (43 samples), Ningxia (39 samples), Tianjin (15 samples), Beijing (25 samples), Inner Mongolia (11 samples), Jiangsu (24 samples), Gansu (13 samples), Hubei (16 samples), Shandong (22 samples) and Anhui (14 samples). The samples were not only collected from clinical dead chickens, but also from suspected sick ones (acting depressed; loss of appetite; emaciation; diarrheal; crippling or growth retardation).

### DNA extraction and viral DNA detection

Clinical samples were oscillated and broken to obtain tissue homogenates as follows: 300 mg of tissues was placed into a 2-mL Eppendorf tube with 500 μL of phosphate-buffered saline (PBS), and two high-pressure steam sterilization small steel balls were added. The solution was oscillated for 3 min twice to break the tissues at a frequency of 29 Hz (MM400, Restch, Germany). Total DNA was extracted from the tissue homogenates using the AxyPrep Body Fluid Viral DNA/RNA Miniprep Kit (Axygen, 15916KC5, USA), following the manufacturer instructions. The DNA was resuspended in nuclease-free water and stored at ‒70°C. Whole DNA was used as template for specific PCR with the CAV-specific primers, CAV-F and CAV-R [16], covering a 582-nucleotide region in the highly conserved overlapping sequence of *VP2* and *VP1*. The PCR aplification was carried out using Premix Ex Taq (Version 2.0 plus dye, TaKaRa Biotechnology Co., Ltd., Dalian, China), MgCl_2_, dNTPs, and 10 pmol each primer in a 20-μL total reaction volume. The reactions ran in an automated thermal cycler (Gene Amp PCR System TC-960F; Blue Marlin, Zurich, Switzerland) under the following program: initial denaturation of 95°C for 5 min, followed by 30 cycles of denaturation at 95°C for 30 s, annealing at 56°C for 30 s, extension at 72°C for 30 s, and final extension at 72°C for 7 min. In all PCR reactions, the CAV strain (Strain M9905)[17] previously isolated and identified in our laboratory was used as the positive control, and the PCR mixture was used as a negative control. The PCR products were analysed by 1.5% agarose gel electrophoresis and imaged using the BIO-RAD Universal Hood II system (USA).

### CAV isolation

MDCC-MSB1 cell cultures are preferred for virus isolation. Freshly prepared cultures containing 2 × 10^5^ cells/well were seeded in 6-well plate. Cells are inoculated with 400uL/well (1:5, v:v) of prepared clinical tissue homogenates. Cultures are split every four days for 10 passages or until cell death is observed[18].

### IFA

IFA was performed using MDCC-MSB1 cells (preserved by our lab) to detect CAV antigens, according to the method described by Yuasa *et al*. [19]. Briefly, CAV-infected cells (PCR positive result) were smeared onto 35-mm culture dishes with 20-mm cover glass inserts (NEST, 801001, China) 3 days post infection, then dried, fixed with 4% paraformaldehyde for 30 min at room temperature (RT), and permeabilised with 0.1% Triton X-100 for 15 min at RT. After three washes with 0.05% Tween 20 PBS (PBST), cells were incubated with mouse monoclonal anti-VP1 primary antibodies (preserved by our lab that produced in mouse by prokaryotic expressed VP1[20]) for 1.5 h at 37°C. After three washes with PBST, cells were incubated for 1 h with a 1:200 dilution of fluorescein isothiocyanate (FITC)-conjugated goat anti-mouse secondary antibody in the dark. After three washes with PBST, the infected MDCC-MSB1 cells were observed by fluorescence microscope (EVOS F1; Life, USA).

### Lysate preparation and Western Blot

The MDCC-MSB1 cells with positive PCR results were harvested by centrifugation, washed with PBS (pH = 7.4), and lysed with lysis buffer containing 20 mM Tris-HCl (pH 7.5), 150 mM NaCl, 1% Triton X-100, sodium pyrophosphate, β-glycerophosphate, and 1× complete cocktail protease inhibitor. Whole lysates were boiled for 10 min in the presence of 5× sodium dodecyl sulfate polyacrylamide gel electrophoresis (SDS-PAGE) loading buffer. After centrifugation at 12,000 × *g* for 2 min, equivalent sample amounts were separated by 12% SDS-PAGE and transferred to pure nitrocellulose blotting membranes (PALL, 66485, USA). After blocking with 5% skim milk, the membranes were incubated with primary antibody at 37°C for 1.5 h, followed by the IRDye 800CW secondary antibody for 1 h at 37°C. Proteins were visualised using the Odyssey system (Li-Cor, USA).

### Amplification of the CAV genome

Two overlapped PCRs were used to map the CAV whole genome. The primers VP1-F (5′-AGCCGACCCCGAACCGCAAGAA-3′) and VP1-R (5′-TCAGGGCTGCGTCCCCCAGTACA-3′) were used to amplify the segment of *VP1*, and the primers InVP1-F (5′-GAGCCGGTAATGAAGAGCGATGCAT-3′) and InVP1-R (5′-CTCCGATGTCGAAATTTATATCTTC-3′) were used to amplify the segment of the complementary gene of *VP1*. We designed our PCR primers based on the reference complete genome sequence of Cux-1. The PCR amplification was carried out in PCR buffer containing 0.4 mM of each dNTP, 10 pmol of each primer, and 2× Phanta Max Master Mix polymerase (Vazyme Biotech Co., Ltd., Nanjing, China) in a 50-μL total reaction volume, using the Gene Amp PCR System TC-960F. These reactions ran under the following program: initial denaturation of 98°C for 5 min, followed by 30 cycles of denaturation at 98°C for 10 s, annealing at 60°C for 15 s, extension at 72°C for 10 s, and final extension at 72°C for 7 min. The PCR products were analysed by 2.0% agarose gel electrophoresis and imaged using the BIO-RAD Universal Hood II system. In all PCR reactions, CAV strain M9905 previously identified in our laboratory was used as a positive control, whereas the PCR mixture was used as a negative control. The two regions (“VP1” and “InVP1”) were purified using the AxyPrep DNA Gel Extraction kit (Axygen, Union City, USA). DNA extraction and PCR were performed at least twice for each sample.

PCR that used to detect other pathogens including MDV (F: 5′-TGCGATGAAAGTGCTATGGAGG-3′; R: 5′-GAGAATCCCTATGAGAAAGCGC-3′), AGV2 (F: 5′-CGTGTCCGCCAGCAGAAAC-3′; R: 5′-GGTAGAAGCCAAAGCGTCCAC-3′)[9], ALV (F: 5′-GGATGAGGTGACTAAGAAAG-3′; R: 5′-CGAACCAAAGGTAACACACG-3′)[21], REV (F: 5′-GCCTTAGCCGCCATTGTA-3′; R: 5′-CCAGCCAACACCACGAACA-3′)[22] and ARV (F: 5′-ATGGCGGGTCTCAATCCATC-3′; R: 5′-TTAGGTGTCGATGCCGGTAC-3′)[23].

### DNA cloning and sequencing

In the case of sequencing failure, specific PCR products of “VP1” and “InVP1” regions were subcloned into the pMD18-T Easy vector (TaKaRa Biotechnology Co., Ltd., Dalian, China), and the ligated products were transformed into *Escherichia coli* DH5α competent cells (Tiangen Biotech Co., Ltd., Beijing, China). Colonies that contained DNA inserts of the correct size were picked and grown overnight in 3 mL of ampicillin-containing lysogeny broth (LB). The mini-preparation of plasmid DNAs was performed with a plasmid extraction kit (AxyPrep Plasmid Miniprep; Union City, USA), following the manufacturer instructions. The plasmid DNAs were sequenced by Comate Bioscience Co., Ltd. (Changchun, Jilin, China).

### Eukaryotic expression of CAV VP1 and transfection

The complete VP1 were cloned into pCAGGS (reserved by our laboratory). The Cux-1 (M55918) viral genes were amplified by polymerase chain reaction (PCR) using the primers wVP1-F (5′-CCGGAATTCCGATGGCAAGACGAGCTCGCAGACCGA-3′) and wVP1-R (5′-CGGGGTACCTCAGGGCTGCGTCCCCAGTACATG-3′). Following purification, the PCR products were digested with the appropriate restriction enzymes and inserted into pCAGGS, generating pCAGGS-wVP1. The plasmid of pCAGGS-wVP1 was transfected into DF-1 cells by Lipofectamine 3000 (Thermo Fisher, CA, US) following the manuals.

### Analysis of sequence data

Contig assembly was performed using Seqman in Lasegene (Dnastar Inc., Madison, WI, USA). Sequences were aligned using ClustalW [24]. Phylogenetic and molecular evolutionary analyses were based on the whole genome, VP1, VP2, and VP3 DNA sequences, using MEGA version 7.0.18 [25]. Phylogenetic analyses of the nucleic acid and deduced amino acid sequences were conducted using the neighbour-joining method [26], the Kimura 2-parameter model [27], pairwise deletion, and 1,000 bootstrap replicates [28]. The new CAV strains were named using the format PPYYXXX, where PP was the location of sampling, and YYXXX was the time and order of the sample origin. Relevant whole genome and coding sequence (CDS) available in GenBank were included for comparison (Table 1).

**Table 1:** The sequence information used in this article.

No.	Isolate	Origin	Year	Accession no.
1	JL14023*	Jilin, China	2014	KY486145
2	JL14024*	Jilin, China	2014	KY486146
3	JL14026*	Jilin, China	2014	KY486147
4	JL14028*	Jilin, China	2014	KY486148
5	HLJ14101*	Heilongjiang, China	2014	KY486136
6	NX15091*	Ningxia, China	2015	KY486150
7	HLJ15108*	Heilongjiang, China	2015	KY486137
8	HLJ15109*	Heilongjiang, China	2015	KY486138
9	JL15120*	Jilin, China	2015	KY486149
10	HLJ15125*	Heilongjiang, China	2015	KY486139
11	NX15140*	Ningxia, China	2015	KY486151
12	JS15165*	Jiangsu, China	2015	KY486152
13	JS15166*	Jiangsu, China	2015	KY486153
14	LN15169*	Liaoning, China	2015	KY486154
15	LN15170*	Liaoning, China	2015	KY486155
16	01–4201	USA	2007	DQ991394
17	3–1	Malaysia	2003	AF390038
18	3–1 P60	Malaysia	2003	AY040632
19	10	United Kingdom	1997	U66304
20	98D02152	USA	2016	AF311892
21	98D06073	USA	2016	AF311900
22	704	Australia	1996	U65414
23	AH4	Anhui, China	2005	DQ124936
24	AH6	Anhui, China	2005	DQ124935
25	BD-3	Germany	2004	AF395114
26	BJ0401	Beijing, China	2005	DQ124934
27	C14	China	2007	EF176599
28	C369	Japan	2001	AB046590
29	26P4	The Netherland	2007	D10068
30	82–2	Japan	2008	D31965
31	CAV-18	Argentina	2014	KJ872514
32	CIA-1	USA	1996	L14767
33	CIAV89-69	South Korea	2011	JF507715
34	Cux-1	Germany	2008	M55918
35	Cuxhaven 1	Germany	1993	M81223
36	G6	Japan	2009	AB119448
37	GD-101	Guangdong, China	2016	KU050680
38	GD-102	Guangdong, China	2016	KU050677
39	GD-103	Guangdong, China	2016	KU050678
40	GD-104	Guangdong, China	2016	KU050679
41	Harbin	Heilongjiang, China	2002	AF475908
42	HN9	Tianjin, China	2005	DQ141672
43	22	Taiwan	2014	KJ728830
44	LF4	Hebei, China	2005	AY839944
45	SD22	Shandong, China	2005	DQ141673
46	SD24	Shandong, China	2005	AY999018
47	SD1403	Shandong, China	2016	KU221054
48	SDLY08	Shandong, China	2008	FJ172347
49	SH11	Shanghai, China	2005	DQ141670
50	SH16	Shanghai, China	2005	DQ141671
51	SMSC-1	Malaysia	2003	AF285882
52	SMSC-1P60	Malaysia	2003	AF390102)
53	TJBD33	Tianjin, China	2005	AY843527
54	TJBD40	Tianjin, China	2004	AY846844
55	TR20	Japan	1999	AB027470

* The strains isolated in this study. The new CAV strains were named using the format PPYYXXX, where PP was the location of sampling, and YYXXX was the time and order of the sample origin.

## Results

### CAV was prevalent in poultry in coinfection type

We obtained 96 CAV-positive samples (13.30%) out of 722 clinical samples, 24 (16.11%) out of 149 being detected in 2014 and 72 (12.57%) out of 573 being detected in 2015. The CAV-positive samples were distributed in all the provinces, including Heilongjiang, Jilin, Liaoning, Hebei, Shanghai, Beijing, Ningxia, Tianjin, Inner Mongolia, Jiangsu, Gansu, and Hubei.

Coinfection was the main infection type of CAV. As shown in Table 2, we found that the single infection of CAV was detected in 32 (41.67%) samples; the remaining samples (58.33%) indicated mixed infection. The 23 dual infections included CAV and the following co-infecting virus: MDV (n = 18), REV (n = 3), AGV2 (n = 1), and ARV (n = 1). The 15 triple infections included CAV and REV + MDV (n = 10), AGV2 + MDV (n = 3), and ARV + MDV (n = 2). Only 2 quadruple infections were identified (CAV with AGV2 + REV + MDV). Mixed infection with MDV was the most common infection type (87.5%) of coinfection samples.

**Table 2:** The CAV infection type of China in 2015 of detected clinical samples.

Infection type		Number of positive	Percentage of positive samples	Percentage of total samples
**Single infection**	CAV	32	41.67%	5.58%
**Dual infection**	CAV+MDV	18	25.00%	3.14%
	CAV+REV	3	4.17%	0.52%
	CAV+AGV2	1	1.39%	0.17%
	CAV+ARV	1	1.39%	0.17%
Subtotal		23	31.94%	4.01%
**Triple infection**	CAV+REV+MDV	10	13.89%	1.75%
	CAV+AGV2+MDV	3	4.17%	0.52%
	CAV+ARV+MDV	2	2.78%	0.35%
Subtotal		15	20.83%	2.62%
**Quadruple infection**	CAV+AGV2 +REV+MDV	2	2.78%	0.35%
Subtotal		2	2.78%	0.35%

### Isolation and sequencing of 15 CAV strains

**Fig 1:**
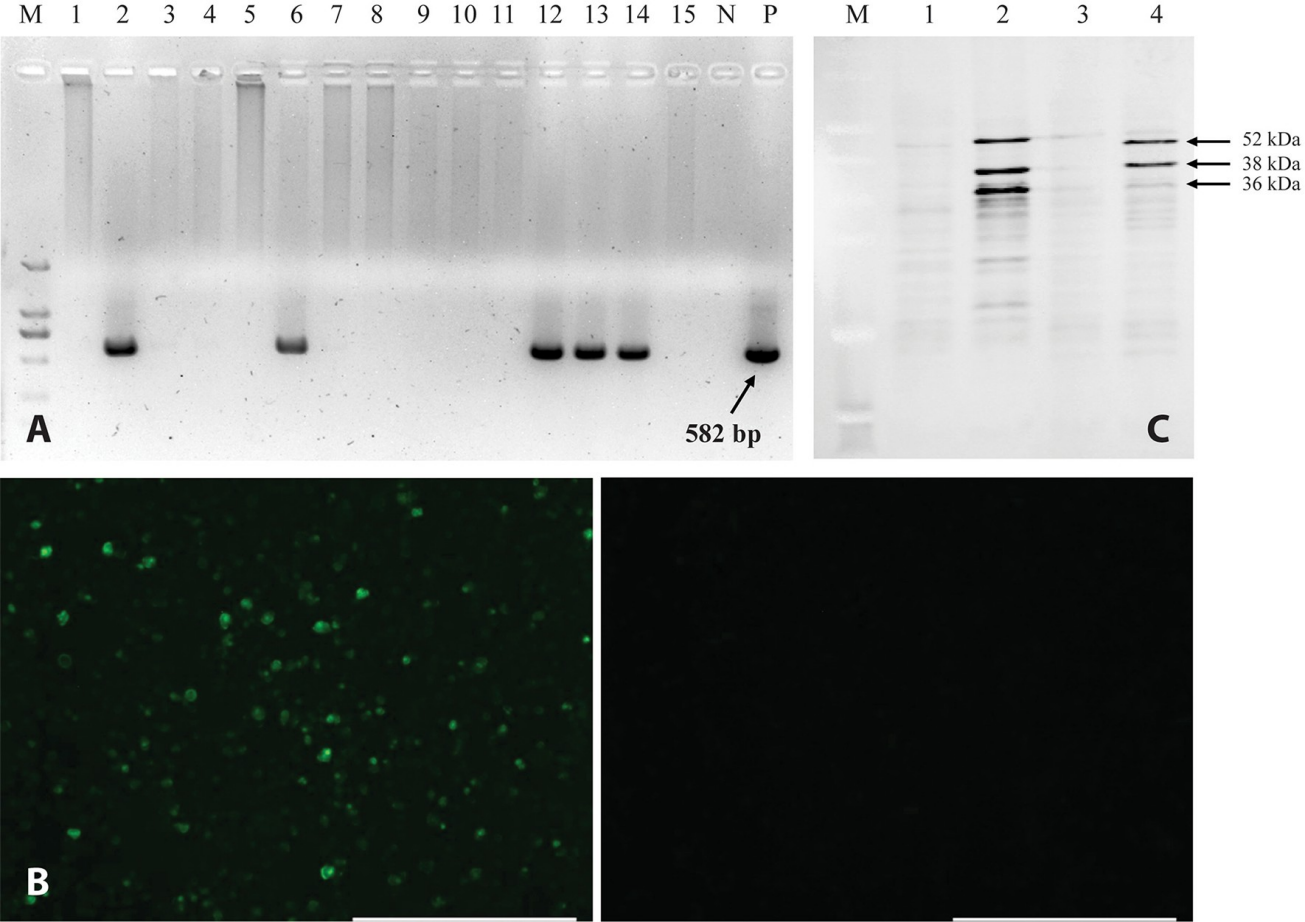
Detection, isolation, and identification of chicken anaemia virus (CAV) from clinical samples. (A) Polymerase chain reaction (PCR) detection of CAV from clinical samples. M, Marker (DNA ladder 2000); N, negative control; P, positive control; Lane 2, 6, 12, 13, and 14 were CAV-positives. (B) Indirect immunofluorescence assay (IFA) result of one of the CAV isolates. The left panel shows MDCC-MSB1 cells infected with CAV isolates, and the right panel shows the Mock control. Size bar = 200 nm. (C) Western Blot analysis of viral protein 1 (VP1) of eukaryotic expression transfected by pCAGGS-wVP1 and MDCC-MSB1 cells infected by isolated virus. M, Marker; Lane 1, Empty Vector of pCAGGS; Lane 2, DF-1 cells transfected by pCAGGS-wVP1, Lane 3, MDCC-MSB1 cells alone; Lane 4, MDCC-MSB1 cells infected by isolated virus of NX15091. Three main bands (52 kDa, 38 kDa, and 36 kDa) are labelled by arrows.

We used polymerase chain reaction (PCR), indirect immunofluorescence assay (IFA) and Western Blot to verify the identification of 15 new CAV strains (5 isolations from 2014 and 10 isolations from 2015) at the nuclei acid, protein, and cell level, respectively after at least 6 passages in MDCC-MSB1 cells. As shown in Fig 1A, DNAs from five CAV-positive MDCC-MSB1 strains were amplified by PCR; Fig 1B shows the green fluorescent signal in infected MDCC-MSB1 cells but no signal in the negative control group. Fig 1C shows the Western Blot result of CAV VP1 of MDCC-MSB1 cells infected by the new CAV strains. Compared to the non-infected cells and the eukaryotic transfection of N-terminal truncated VP1, three main bands were observed. One 52-kDa band (50-kDa of the truncated) was observed as expected, two additional bands (38-kDa and 36-kDa band) were also detected.

The whole genomic sequences of each strain were deposited in GenBank under accession numbers KY486136-KY486139 and KY486145-KY486155. All the detailed information of the new sequences and the reference sequences are shown in Table 1.

### Phylogenetic analysis of CAV genome sequences

The 15 new CAV genomes had a length of 2,298 nucleotides, containing only four 21-nucleotide direct repeat regions in the 5'-untranslated regions (UTRs). All the sequences had high levels of nucleotide similarity to the reference strain Cux-1 (Germany, M55918), with NX1501 having the lowest level of nucleotide similarity (97.4%) and the strains HLJ14101 and HJ15125 having the highest level of nucleotide similarity (98.0%). The obtained sequences had a high level of nucleotide similarity with each other, the divergence is only 1.0% (JS15165 and JS15166) to 2.5% (HLJ1508 and JL15120).

**Fig 2:**
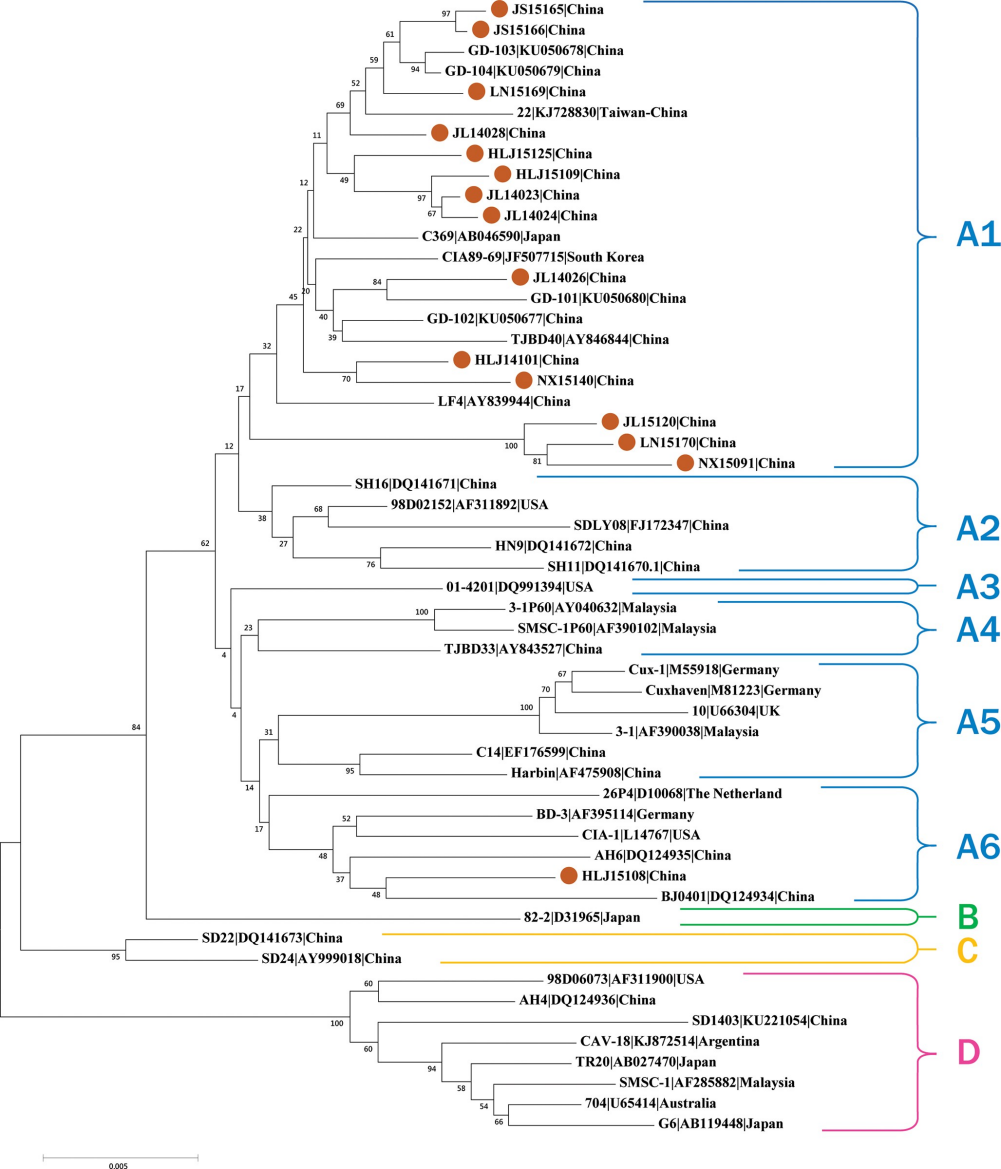
Phylogenetic analysis of the nucleotide sequences of 15 new complete genomes and other 40 complete genomes available in GenBank. Sequences from the present study (brown closed circle) are named as mentioned in the main text. Sequences from GenBank are named by the country name followed by accession number. The four major groups were identified as A, B, C, and D as well as subgroups A1, A2, A3, A4, A5, and A6 in blue, green, yellow, and red, respectively. The percentages of replicate trees in which the associated taxa clustered together in the bootstrap test (1,000 replicates) are shown next to the branches.

Phylogenetic analysis at the nucleotide level of the 15 new CAV genome sequences together with 40 reference sequences including Cux-1, CIA-1 (USA, L14767), TR20 (Japan, AB027470), SMSC-1 (Malaysia, AF285882), and 3–1 (Malaysia, AF390038 was conducted. All sequences were clustered into four distinct sequence groups (A, B, C, and D), with bootstrap values of 84, 95, and 100, respectively (Fig 2). In addition, Group A comprised of six distinct subgroups based on the shape of tree branches. The Asian sequences isolated from mainland China, Taiwan, Japan, and South Korea belonged to Subgroup A1. Four Chinese sequences and the American sequence 98D02152 (AF311892) were the members of Subgroup A2. Subgroup A3 contained only one sequence (01–4202 (DQ991394)). Subgroup A4 comprised two Malaysian and one Chinese strain. Cux-1, two European sequences, and two Chinese sequences constituted Subgroup A5. CIA-1, other two European sequences, and three Chinese sequences belonged to Subgroup A6. Group B comprised only one sequence (82–2 (D31965)), and Group C comprised two Chinese strains (SD22 (DQ141673) and SD24 (AY999018)). Group D consisted of various region sourced sequences including strains from North America, South America, Australia, and Asia. Overall, all the sequences in this study belonged mainly to Subgroup A1 (14/15, 93.33%), indicating that the main CAV prevalent isolates possessed the typical Asian virus characteristics in whole genome sequence, except strain HLJ15108 (1/15, 6.67%) which belonged to Subgroup A6 with a unique mutation (Fig 2).

### The diversity of VP1 and the conservatism of VP2 and VP3

Compared to all the reference sequences, 449 aa-VP1 showed high diversity. We detected 59 substitutions in the VP1 amino acid sequences (amino acid sites 2, 14, 21, 22, 29, 41, 51, 75, 76, 82, 83, 92, 97, 98, 125, 139, 140, 141, 144, 150, 151, 157, 172, 183, 193, 198, 202, 207, 211, 224, 229, 240, 245, 248, 251, 254, 257, 265, 267, 270, 287, 290, 294, 297, 315, 321, 362, 370, 375, 376, 394, 413, 427, 433, 436, 444, 446, 447, and 448), with a mutation probability of 13.14%. Twenty-three substitutions were detected in VP2 (amino acid sites 5, 6, 28, 35, 43, 46, 60, 70, 74, 77, 116, 126, 143, 153, 160, 163, 167, 177, 180, 186, 187, 201, and 209), with a mutation probability of 10.65%. Twenty-two substitutions were identified in VP3 (amino acid sites 2, 3, 4, 8, 12, 21, 25, 31, 39, 42, 54, 64, 67, 70, 73, 81, 91, 103, 105, 108, 116, and 118), with a mutation probability of 18.18%. However, nine amino acids sites (22, 141, 151, 229, 254, 287, 294, 370, and 447) in VP1 had at least three substitutions each, while there was only one amino acid site (28) in VP2 and two amino acid sites (25 and 81) in VP3.

**Fig 3:**
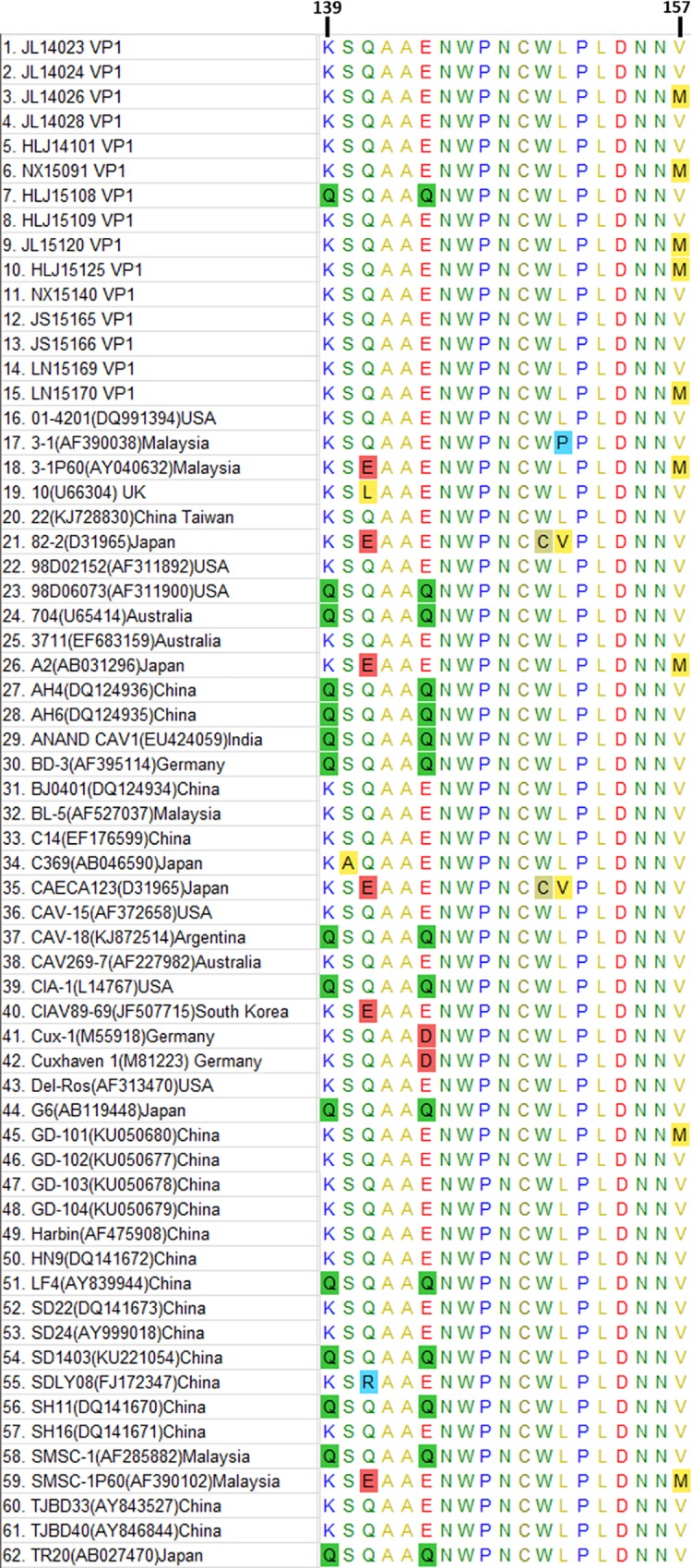
Amino acid alignments of the hypervariable region of different chicken anaemia virus (CAV) viral protein 1 (VP1) coding sequences (aa 139–157). Sequences were compared with each other, and amino acids were indicated by a single-letter code. The substitutions of amino acids were highlighted by the colour of background where occurred at sites 139, 140, 141, 144, 150, 151, and 157.

In the hypervariable region (amino acid sites 139 to 157) of the VP1 sequence (Fig 3), the new strains, except HLJ15108, manifested the presence of lysine (K) at site 139 and glutamic acid (E) at site 144 instead of glutamine (Q). However, five sequences (JL14026, NX15091, JL15120, HLJ15125, and LN15170) had a methionine (M) substitution for valine (V) at site 157.

**Fig 4:**
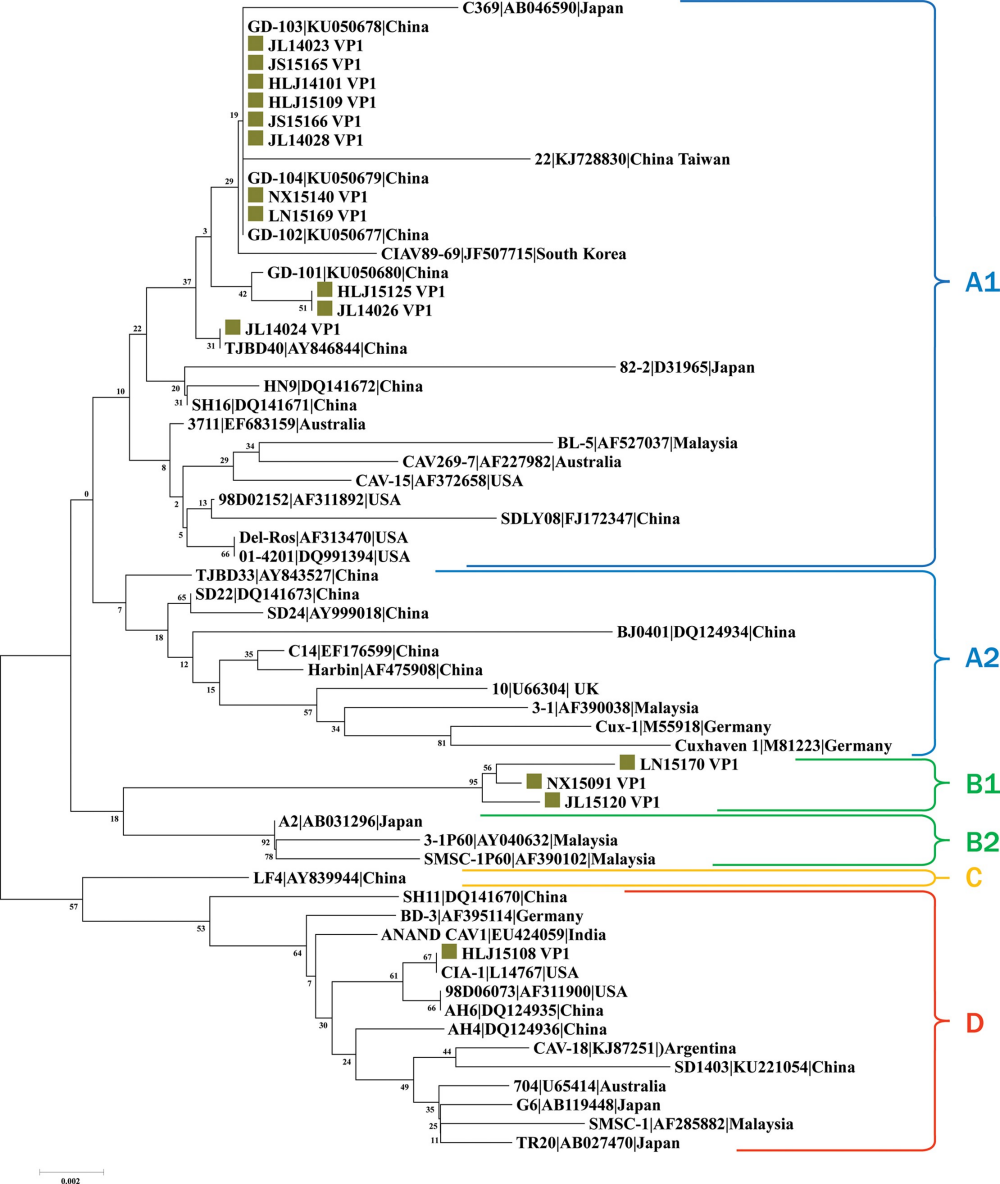
Phylogenetic analysis of the amino acid sequences of 15 new viral protein 1 (VP1) sequences and other 46 VP1 sequences available in GenBank. Sequences in this study (green closed square) are named as mentioned in the main text. Sequences from GenBank are named by the country name followed by the accession number. The four major groups were identified as A, B, C, and D as well as subgroups A1, A2, B1, and B2 in blue, green, yellow, and red, respectively. The percentages of replicate trees in which the associated taxa clustered together in the bootstrap test (1,000 replicates) are shown next to the branches.

Four major groups were present in the VP1 protein sequence phylogenetic tree (Fig 4). Strains JL14023, JS15165, HLJ14101, HLJ15109, JS15166, JL14028, NX15140, LN15169, HLJ15125, JL14026, and JL14024 clustered in Subgroup A1. Six Chinese strains, the Malaysian 3–1, and Cux-1 belonged to Subgroup A2. Subgroup B1 only contained three new sequences (LN15170, NX15091, and JL15120). Subgroup B2 contained three reference strains from Asia. Group C contained only one sequence from China. Group D contained one new sequence (HLJ15108) and various region sourced sequences.

Unlike VP1, VP2 and VP3 protein sequences were conservative. All the sequences clustered in one main group (S1 and S2 Figs).

### The critical amino acid substitution in CAV VP1

At the amino acid level, the new CAV strains showed virulent characteristics (Table 3). At amino acid sites 89, 141, and 394, all CAV isolates presented threonine (T) instead of alanine (A), typical of low virulent strains. At amino acid site 75, fourteen new isolates presented valine (V), except HLJ1508 that presented isoleucine (I), as low virulent strains. At amino acid sites 125, 139, and 144, fourteen isolates presented leucine (L), lysine (K), and glutamic acid (E), respectively, as low virulent strains, but the substitutions in HLJ1508 were isoleucine (I), glutamine (Q) and glutamine (Q) as the majority of virulent strains. The substitution at amino acid site 157 involved five strains; in JL14023, JL14024, JL14028, HLJ14101, HLJ15108, HLJ15109, NX15140, JS15165, JS15166, and LN1516 the substitution was valine (V) the majority of virulent strains, while in JL14026, NX15091, JL15120, HLJ15125, and LN15170 the substitution was methionine (M) as low virulent strains. In addition, substitutions at the amino acid site 287 were observed; thirteen strains had a serine (S), as low virulent strains, LN15170 had a threonine (T), as the majority of virulent strains, however, HLJ1508 had an alanine (A) that has not been identified in other strains.

**Table 3:** The key amino acids of VP1 determines the virulence of CAV.

CAV Strain	Amino acid								
Amino acid position	75	89	125	139	141	144	157	287	394
**Majority virulent strain**	**V**	**T**	**I**	**Q**	**Q**	**Q**	**V**	**T**	**Q**
**Low virulent strain**	**I**	**A**	**L**	**K**	**E**	**E**	**M**	**S**	**H**
**JL14023**	**V**	**T**	**L**	**K**	**Q**	**E**	**V**	**S**	**Q**
**JL14024**	**V**	**T**	**L**	**K**	**Q**	**E**	**V**	**S**	**Q**
**JL14026**	**V**	**T**	**L**	**K**	**Q**	**E**	**M**	**S**	**Q**
**JL14028**	**V**	**T**	**L**	**K**	**Q**	**E**	**V**	**S**	**Q**
**HLJ14101**	**V**	**T**	**L**	**K**	**Q**	**E**	**V**	**S**	**Q**
**NX15091**	**V**	**T**	**L**	**K**	**Q**	**E**	**M**	**S**	**Q**
**HLJ15108**	**I**	**T**	**I**	**Q**	**Q**	**Q**	**V**	**A**	**Q**
**HLJ15109**	**V**	**T**	**L**	**K**	**Q**	**E**	**V**	**S**	**Q**
**JL15120**	**V**	**T**	**L**	**K**	**Q**	**E**	**M**	**S**	**Q**
**HLJ15125**	**V**	**T**	**L**	**K**	**Q**	**E**	**M**	**S**	**Q**
**NX15140**	**V**	**T**	**L**	**K**	**Q**	**E**	**V**	**S**	**Q**
**JS15165**	**V**	**T**	**L**	**K**	**Q**	**E**	**V**	**S**	**Q**
**JS15166**	**V**	**T**	**L**	**K**	**Q**	**E**	**V**	**S**	**Q**
**LN15169**	**V**	**T**	**L**	**K**	**Q**	**E**	**V**	**S**	**Q**
**LN15170**	**V**	**T**	**L**	**K**	**Q**	**E**	**M**	**T**	**Q**

## Discussion

In this research, we investigated the mixed infection situation of CAV and other common immunosuppressive diseases in the chosen provinces of China that was rarely reported. Moreover, we analysed the sequences compared with the reported information that covered a more extensive strain distribution than ever before. Based on our investigation, a high positive rate (13.30%) of CAV was detected in sick chickens. Especially, the morbidity and mortality were considerably enhanced when chicks are dually infected with CAV and MDV, REV, or infectious bursal disease virus (IBDV), probably because of the virus-induced immunosuppression [29–33]. Talbe1 showed the infection type of samples collected in 2015, out of all the 72 CAV-positive samples, there were 35 (48.6%) samples were detected as MDV positive (18 dual infection; 15 triple infection; 2 quadruple infection), but other viral pathogens showed a similar ratio that was not particularly high, therefore, meaning MDV and CAV were the most common co-infection type in this investigation, as believed that outbreaks of MD in birds most are linked with CAV co-infection [34], which should arouse more attention. It is known that with CAV infected commercial chickens, lymphocytic depletion of the bursa often leads to lymphocytic depletion of the thymus [35, 36]. Therefore, in case of commercial flocks, immunosuppression by alternative viral agents, such as IBDV or MDV, is crucial in the outcome of CAV infection. Certain strains of reovirus can be immunosuppressive in chickens [37], which demonstrates the reovirus has strong link to the pathogenicity of CAV [37].

VP1, the only structural protein in CAV, encodes the viral capsid, whose predicted mass is 50 kDa [38]. Interestingly, in this study, two additional VP1-specific bands (38 kDa and 36 kDa) were detected (Fig 1). These novel naturally truncated forms of VP1 may be generated by enzymatic digestion, playing important function(s) required for the viral lifecycle. Similar phenomena have been observed in various other viruses, including herpes simplex virus (HSV-1), Aleutian mink disease virus (AMDV) [39, 40], hepatitis C virus (HCV) [41], adenovirus [42], influenza A virus [43], and severe acute respiratory syndrome (SARS) virus [44]. Certain sites induced caspase-dependent cleavage to benefit viral replication. Currently, we are attempting to identify the peptide cleavage sites and the function of the derived peptides.

Partial or complete genome sequences of numerous strains from different parts of the world have been determined. Amino acid sequences have been predicted as well. With most of the variation in the VP1 coding region, generally less than 5% of limited genetic variation is known among isolates [3, 45–47]. In the case of predicted amino acid sequences, minor differences have been noted particularly in the VP1 amino acid sites 139–151. The observed differences in the hypervariable region had impact on the predicted protein structure of VP1 [48–50]. In this study, the phylogenetic analysis of VP1 embodied the diversity of CAV, while the VP2 and VP3 embodied the conservatism. The non-structural VP2 protein probably acts as a scaffold protein during virion assembly, ensuring that VP1 folds correctly [51, 52], which can also induce apoptosis [53]. VP3, also known as apoptin [54]. Based on its ability to mediate cell death selectively in cells that have encounter an oncogenic transformation, VP3 has drawn a lot of attention [55, 56]. The localization of VP3 in infected cells are affected by presented threonine (T), which may be phosphorylated, at amino acid sites 20, 56, 61 and 114 [57]. In our study, we detected T residues in all the sequences, indicating conservatism that may contribute to the function of these two non-structural proteins and to the lifecycle of CAV.

The amino acid site 394 in VP1 was reported to be a major genetic determinant of virulence [16]. CAV strains are highly pathogenic if glutamine (Q) is present or less pathogenic if histidine (H) is present [6, 58, 59]. All the new isolates in this study had glutamine (Q) at amino acid site 394, indicating that all strains were highly pathogenic. At amino acid site 89, the attribution to the VP1 amino acid is due to the pathogenicity and monoclonal antibody reactivity differences between two molecularly cloned and highly passaged CAV strains [53]. The study of other mutations (I75, L125, E141, and E144) regarding attenuation has been reported. Renshwa et al. [40] proposed that Q139 and/or Q144 has influence on the rate of replication or dissemination of infection in MDCC-MSB1 cells. von Bülow and Fuchs [55] stated the attenuation of Cux-1 was found after passages (P49) in cell culture. Whereas according to Todd et al. [56], Cux-1 became considerably less pathogenic at P173 in MDCC-MSB1 cells. As reported by Chowdhury et al. [52], two Malaysian CAV strains were partly attenuated at p6 and one was further attenuated at P123 in MDCC-MSB1 cells. Both 3-1P60 (AY040632) and SMSC-1P60 (AF390102) had substitutions M157 and S287. The characteristics of the critical amino acid residues in the VP1 from the new isolates in this study indicated that although almost all the strains were virulent, some had the features of low virulent or attenuated viruses. Therefore, in this study the CAV with low or attenuated virulent features were probably mixed as a contaminant in various vaccines [8], and recover their virulence in the host. Consequently, monitoring and improving the quality of vaccines may be a useful way to control the prevalence of CAV. Using high quality chicks and embryos (CAV-free) in the vaccines production process will be a good option. Furthermore, it is necessary to eliminate and replace the CAV positive chickens.

## Supporting information

S1 Fig Phylogenetic analysis of the amino acid sequence of 15 new viral protein 2 (VP2) sequences and other 47 VP2 sequences available in GenBank. Sequences in this study (blue-green closed triangle) are named as mentioned in the main text. Sequences from GenBank are named by the country name followed by the accession number. The percentages of replicate trees in which the associated taxa clustered together in the bootstrap test (1,000 replicates) are shown next to the branches.
(TIF)

S2 Fig Phylogenetic analysis of the amino acid sequence of 15 new viral protein 3 (VP3) sequences and other 47 VP3 sequences available in GenBank. Sequences in this study (red closed diamond) are named as mentioned in the main text. Sequences from GenBank are named by the country name followed by the accession number. The percentages of replicate trees in which the associated taxa clustered together in the bootstrap test (1,000 replicates) are shown next to the branches.
(TIF)
