# Supplementary figures and images for: Molecular epidemiology of chicken anaemia virus in sick chickens in China from 2014 to 2015
Source: PLoS One. 2019 Jan 18;14(1):e0210696. doi: 10.1371/journal.pone.0210696 (PMC6338413; doi:10.1371/journal.pone.0210696)

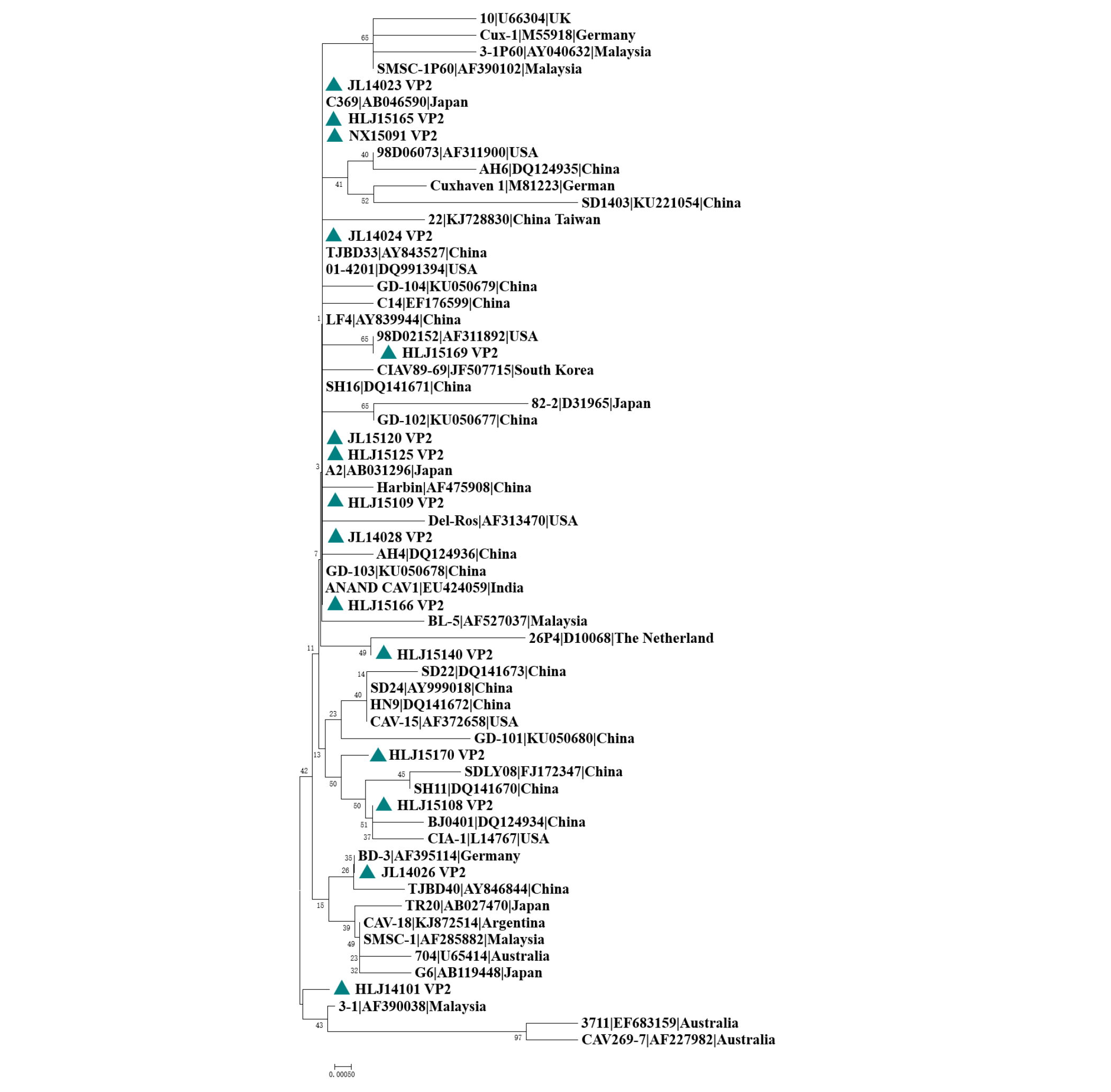

Supplement: S1 Fig — Sequences in this study (blue-green closed triangle) are named as mentioned in the main text. Sequences from GenBank are named by the country name followed by the accession number. The percentages of replicate trees in which the associated taxa clustered together in the bootstrap test (1,000 replicates) are shown next to the branches. (TIF) [file pone.0210696.s001.tif]

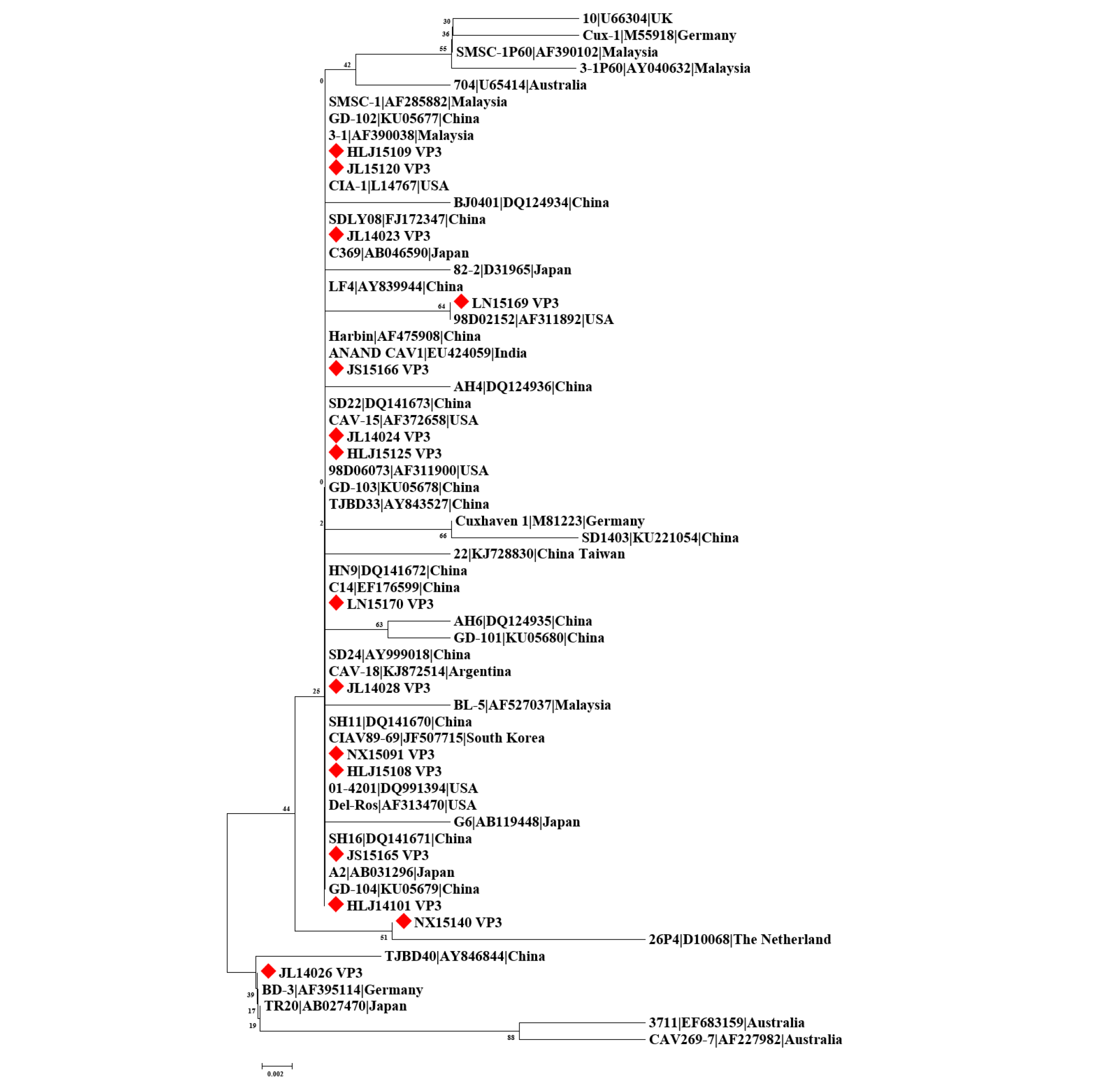

Supplement: S2 Fig — Sequences in this study (red closed diamond) are named as mentioned in the main text. Sequences from GenBank are named by the country name followed by the accession number. The percentages of replicate trees in which the associated taxa clustered together in the bootstrap test (1,000 replicates) are shown next to the branches. (TIF) [file pone.0210696.s002.tif]
